# Northward dispersal of sea kraits (*Laticauda semifasciata*) beyond their typical range

**DOI:** 10.1371/journal.pone.0179871

**Published:** 2017-06-23

**Authors:** Jaejin Park, Il-Hun Kim, Jonathan J. Fong, Kyo-Soung Koo, Woo-Jin Choi, Tein-Shun Tsai, Daesik Park

**Affiliations:** 1 Department of Biology, Kangwon National University, Chuncheon, Kangwon, South Korea; 2 Science Unit, Lingnan University, Tuen Mun, New Territories, Hong Kong; 3 Department of Biological Science and Technology, National Pingtung University of Science and Technology, Neipu Township, Pingtung County, Taiwan (Republic of China); 4 Division of Science Education, Kangwon National University, Chuncheon, Kangwon, South Korea; Centre National de la Recherche Scientifique, FRANCE

## Abstract

Marine reptiles are declining globally, and recent climate change may be a contributing factor. The study of sea snakes collected beyond their typical distribution range provides valuable insight on how climate change affects marine reptile populations. Recently, we collected 12 *Laticauda semifasciata* (11 females, 1 male) from the waters around southern South Korea—an area located outside its typical distribution range (Japan, China including Taiwan, Philippines and Indonesia). We investigated the genetic origin of Korean specimens by analyzing mitochondrial cytochrome b gene (*Cytb*) sequences. Six individuals shared haplotypes with a group found in Taiwan-southern Ryukyu Islands, while the remaining six individuals shared haplotypes with a group encompassing the entire Ryukyu Archipelago. These results suggest *L*. *semifasciata* moved into Korean waters from the Taiwan-Ryukyu region via the Taiwan Warm Current and/or the Kuroshio Current, with extended survival facilitated by ocean warming. We highlight several contributing factors that increase the chances that *L*. *semifasciata* establishes new northern populations beyond the original distribution range.

## Introduction

Reptiles, both terrestrial [1] and marine [2–4], are declining on a global scale. The major contributing factors for marine reptile declines include climate change, deterioration of habitat quality and overexploitation [5,6]. In particular for marine reptiles, ocean warming due to climate change could widely affect breeding patterns, abundance and distribution [2,7]. To understand how climate change affects marine reptile populations, data on changes in geographic distribution (e.g. records of individuals beyond the typical range) are useful.

In this study, we focus on sea snakes. For marine reptiles, sea turtles are the common example for population declines [3], but sea snakes are also decreasing [3,8,9]. Sea snakes primarily inhabit tropical and subtropical regions of the Indian and Pacific Oceans and consist of two major groups: true sea snakes (Hydrophiinae), which exclusively use marine environments, and sea kraits (Laticaudinae), which use both marine and terrestrial habitats [10,11]. Based on recent reports, sea snake captures outside the typical distribution range are increasing: *Hydrophis platurus* from California, USA [12], *Laticauda semifasciata* and *L*. *laticaudata* from mainland Japan [13], *L*. *semifasciata* and *H*. *platurus* from Russia [14] and *L*. *semifasciata* from Korea [15–17]. It has been suggested that these new records are a result of ocean warming following recent climate change.

Historically, only three Hydrophiine species (*H*. *platurus*, *H*. *cyanocinctus*, *H*. *melanocephalus*) were reported from Korea [18–21]. The capture of *L*. *semifasciata* in Korean waters represents a new and recent appearance [15–17]; *Laticauda semifasciata* is typically found in the waters around Japan, China including Taiwan, Philippines and Indonesia [22]. Tandavanitj et al. [23] found that despite its dispersal abilities, *L*. *semifasciata* showed distinctive genetic structure between island groups in Taiwan and Japan. In this study, we use molecular methods to determine the geographic origin and cause of *L*. *semifasciata* recently collected from Korean waters.

## Materials and methods

### Collection of sea snake samples

Animal handling and experimental procedures were conducted in accordance with guidelines established by the Kangwon National University Institutional Animal Care and Use Committee (Permit Number: KW-161108-1). *Laticauda semifasciata* is not protected in South Korea, so the collection of sea snakes does not require a permit. The individuals were caught from open waters, which are not privately owned or protected.

To obtain sea snake samples from Korean waters, we placed more than 200 posters and 50 banners around coastal towns of southern South Korea bordering the South Sea (including Jeju Island) between April 2015 and October 2016. A total of 12 *L*. *semifasciata* (six dead, six alive) were collected and donated by local fishermen—nine from Jeju Island and three from the Korean Peninsula. For convenience, we named the locality based on the nearest port to the capture site. All specimens were delivered to the Herpetological Lab of Kangwon National University (KNU). Upon arrival, we collected basic data on the specimens: sex (using a probe), snout-vent length (SVL; using a tape measure up to 0.1 cm) and body weight (using a digital balance up to 0.1 g; ELT 4001, Sartorius-Korea, Seoul Korea). For tissue collection, we took a tail clip (usually only the terminal scale) using scissors [24]. Additional tail tissue samples from four *L*. *semifasciata* specimens (from Orchid Island, Taiwan) were included in our study to increase geographic sampling. Sequences from the Ryukyu Islands, Japan were obtained from GenBank [23].

### DNA extraction and PCR

We extracted whole genomic DNA from tissue samples using the QIAGEN DNeasy Blood & Tissue kit (QIAGEN, Hilden, Germany) following the manufacturer’s instructions. For this study, we targeted *Cytb* because there are comparable data in GenBank. We amplified partial sequences of the *Cytb* gene using the primers L14910 and H16064 [25]. DNA was amplified using a SimpliAmp Thermal Cycler (Life Technologies, Carlsbad, CA, USA) in 25 μl reaction volumes, consisting of 10 ng of template DNA, 1.25 U of ELPIS rTaq DNA polymerase (ELPIS, Daejeon, South Korea), 2.5 μl of 10x PCR buffer, 2 μl of 10 mM dNTP mix (2.5 mM each) and 0.5 μl of each primer (10 pmol). The cycling conditions for PCR were as follows: 94°C for 4 min, followed by 35 cycles at 94°C for 30 s, 57°C for 30 s and 72°C for 1 min with a final extension step of 72°C for 7 min [25]. We verified PCR products by electrophoresis on 1.5% agarose gels, and purified products using an AccuPrep^®^ PCR Purification Kit (Bioneer, Daejeon, South Korea). PCR products were sequenced in both directions using the same PCR primers at Macrogen (Seoul, South Korea) on a 3730xl DNA analyzer (Applied Biosystems, Foster City, CA, USA).

### Sequence data analysis

We edited and assembled sequences using Geneious v5.3.6. To verify our specimens were all *L*. *semifasciata*, we constructed a phylogenetic tree using 16 new sequences (GenBank accession numbers KY445753 to KY445768), 11 *Laticauda* sequences from GenBank (5 of *L*. *semifasciata*, 4 of *L*. *laticaudata* and 2 of *L*. *colubrina*) and 3 *Hydrophis* sequences as outgroups. (S1 Fig). Sequences were aligned using MUSCLE [26], and the alignment was analyzed using both maximum likelihood (ML) and Bayesian inference (BI) methods. ML analyses were run in RAxML v8.2.4 [27] inferring the best-scoring ML tree with 100 replicates followed by a nonparametric bootstrap analysis of 1000 replicates evaluate node robustness of the ML tree. All replicates were run under the GTR + gamma model of sequence evolution. For BI analyses, Markov Chains Monte Carlo chains were run for 2 million generations, sampling every 1000 generations, implemented in MrBayes v.3.2.4 [28]. Models of nucleotide substitution were chosen within MrBayes using the reversible jumping model choice (nst = mixed) with both rate variation and invariable sites (rates = invgamma). Stationarity was checked graphically by plotting log-likelihood scores in Tracer v.1.5 (http://tree.bio.ed.ac.uk/software/tracer). The first 500,000 generations were discarded as burn-in and the remaining trees were used to build a consensus tree.

For population genetic analyses, we built a haplotype network using a median joining method in PopArt 1.7.2 (http://popart.otago.ac.nz). The dataset analyzed is a combination of our data (16 individuals) with data from Tandavanitj et al. [23] (16 haplotype sequences from 177 individuals).

## Results

Of the 12 snakes from Korea, 11 individuals were female and one was male. Detailed morphological information of each snake is provided in S1 Table.

The phylogenetic trees inferred using ML and BI were highly similar, only differing by support values and the relationships of some terminal branches. Phylogenetic analyses verified the identity of the 16 new specimens (12 Korea, 4 Taiwan) to be *L*. *semifasciata* (S1 Fig).

**Fig 1:**
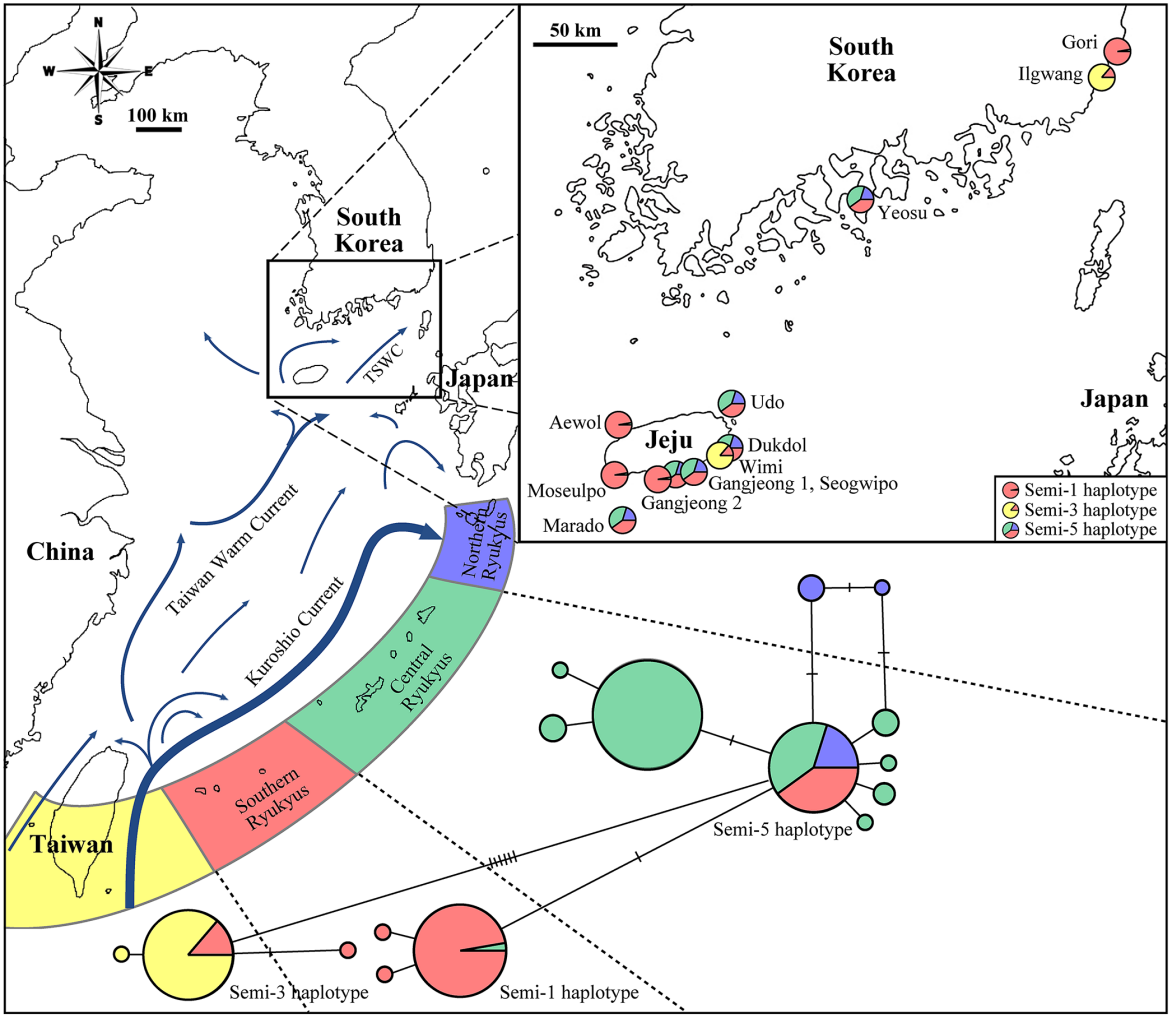
Haplotype network (*Cytb*) of *Laticauda semifascaita* from East Asia. The four major geographic areas in Taiwan-Ryukyu Archipelago (Taiwan, southern, central and northern Ryukyu) are color-coded. Each circle in the network represents a haplotype, and the color represent the geographic origin, while the size represents the frequency of the haplotype. For clarity, we only label three haplotype names, which were found in new specimens examined in this study. The map of Korea indicates the location of each individual, as well as its matching haplotype in Taiwan and the Ryukyu Archipelago. Arrows indicate the path of the major ocean currents in the region. TSWC; Tsushima Warm Current.

We identified three different *Cytb* haplotypes from our specimens, all which were previously reported from the Taiwan-Ryukyu Archipelago (Fig 1); we adopted the same haplotype names as the previous study [23]. Three specimens from Aewol, Moseolpo, and Gangjeong 2 (Jeju Island) and one from Gori (Korean Peninsula) had the Semi-1 haplotype, a haplotype primarily from the southern Ryukyu Islands (37 out of 38 specimens; [23]). One specimen from Wimi (Jeju Island), one from Ilgwang (Korean Peninsula), and four from Taiwan had the Semi-3 haplotype, a haplotype from Taiwan and the southern Ryukyu Islands (27 from Taiwan and 5 from southern Ryukyus; [23]). The five remaining specimens from Jeju Island (Marado, Gangjeong 1, Seogwipo, Dukdol, and Udo) and one from the Korean Peninsula (Yeosu) had the Semi-5 haplotype, a haplotype found throughout the Ryukyu Archipelago (Fig 1).

The 12 Korean samples had only three haplotypes (Semi-1, 3 and 5), which could be placed into two major groups based on geography: (1) Semi-1 and Semi-3 haplotypes originating from Taiwan-southern Ryukyu (Aewol, Moseulpo, Gangjeong 2, Gori, Wimi, Ilgwang) and (2) Semi-5 haplotype originating from the entire Ryukyu Archipelago (Yeosu, Udo, Dukdol, Gangjeong 1, Seogwipo, Marado). Korean samples did not have any haplotypes restricted to either central or northern Ryukyus.

## Discussion

We identify two genetic groups of *L*. *semifasciata* from Korean waters: one group (6 individuals) is closely related to the entire Ryukyu Archipelago (Semi-5), while the second group (6 individuals) has genetic affinity to Taiwan and the southern Ryukyu Islands (Semi-1, Semi-3). For the first group, there is not enough genetic structure to infer the specific geographical origin beyond the Ryukyu Islands. However, for the second group, the available data suggest that the genetic origin of these individuals is either Taiwan or the southern Ryukyus. It is possible, based on the pathways of the Kuroshio Current [29], that the origin of Korean individuals is from further south (Philippines and Indonesia), but genetic data from this region are needed to evaluate this possibility. Ocean currents play a key role in dispersal genetic differentiation in various marine animals, through allowing passive drifts and active swimming of animals in the currents [30–32], and the Taiwan Warm Current and the Kuroshio Current seem to be important for northward dispersal of *L*. *semifasciata*. The presence of *L*. *semifasciata* in Korea was previously undetected, but becoming more common, and we believe that these currents along with recent climate change have contributed to increased records.

In the past, only Hydrophiine species were known to occur in Korea, with fewer than seven observations [20,21], while the first record of Laticaudine sea snakes was in 1995 [16]. The frequency of *L*. *semifasciata* sightings has increased, while *H*. *platurus* observation and capture has been relatively steady [17,33]. To explain the occurrence of *L*. *semifasciata* in Korean waters, we consider three contributing factors: 1) increased effort, 2) northward expansion of prey fish and 3) higher ocean temperatures in Korean waters.

First, increased observation of Laticaudine snakes may be attributed to increased effort in sea snake research relative to the past. We do not believe this to be a major factor because increased effort should also increase observations of Hydrophiine sea snakes known to exist in Korea; during our study period, we had only two reports of *H*. *platurus*, while we collected 12 *L*. *semifasciata* and had greater than six observations. The increased numbers of *L*. *semifasciata* in Korean waters is likely a true pattern.

Second, the northward expansion of prey fish species due to climate change in the East China Sea might increase survival of the sea kraits in northern areas by providing suitable prey. Previous studies showed that global climate change actively or passively expanded various marine fish species beyond their typical distribution ranges [34–36]. In the waters around Jeju Island, subtropical and tropical fish are being captured more frequently [37,38], such as *Chromis notatus* and *Haliechoeres tenuispinis* (family Labridae). Previous studies found *L*. *semifasciata* consume these two fish species [39,40]. So, when *L*. *semifasciata* are in waters around Korea, they can feed on familiar prey fish, resulting in longer survivorship and more sea krait records.

Third, higher ocean temperatures could contribute to survival of *L*. *semifasiata* in northern waters. In such a situation, *L*. *semifasciata* are occasionally dispersed northward, but it is only recently with warmer temperatures that individuals could survive, at least temporarily. The annual mean ocean surface temperature around Jeju Island has increased at a rate of 0.024°C/ year (from 17.9°C to 18.6°C) between 1971 and 2000. Winter temperatures are colder and more critical for survival, and these temperatures have also increased (December: 15.41 to 16.52°C; February: 12.71 to 13.32°C) [41]. This suggests that *L*. *semifascaita* that moved into Korean waters should survive for a longer period than previous, resulting in increased observation of sea kraits.

Our study raises two additional questions: (1) why was *L*. *semifasciata* the only collected sea krait species? and (2) why were more female *L*. *semifasciata* captured? We provide potential explanations for these questions. There are three *Laticauda* species found in Northeast Asia: *L*. *semifasciata*, *L*. *colubrina* and *L*. *laticaudata* [42–44]. The capture of only *L*. *semifasciata* in Korean waters might be due to the geographic distribution, habitat use and physical characteristics of the three species. At a global scale, *L*. *colubrina* and *L*. *laticaudata* have a wide regional distribution throughout Southeast Asia, whereas *L*. *semifasciata* is has a more restricted, northeastern distribution (Taiwan, the Ryukyu Islands, Philippines and Indonesia) [22,45]. The core and largest populations of *L*. *semifasciata* are located in Taiwan and the Ryukyu Islands [22]. If the Taiwan Warm Current and the Kuroshio Current play a major role in northward dispersal, *L*. *semifasciata* should have the highest chance to move into Korean waters. In addition, of the three *Laticauda* species, *L*. *semifasciata* is the most adapted to marine environments: spends the more time in the water [10,45,46], has the lowest net water loss [47] and has a wider tail and more cylindrical body trunk [46]. These factors increase the chance that *L*. *semifasciata* drifts or moves into ocean currents.

Why were more female *L*. *semifasciata* captured? Of the 12 total sea kraits collected in this study, 11 sea kraits were female (92%). We hypothesize that different feeding behavior and abilities contribute to the skew of females found in Korean waters. *Laticauda* species are benthic feeders and often make trips to the deep bottom (> 80 m) of the ocean for an extended time (> 130 min) to obtain their food [48,49]. Because of a larger body size [50], females need larger and more prey items, and likely have better diving performance (deeper and for longer duration) [49]. Such foraging patterns would result in female *L*. *semifasciata* more frequently drifting into the rapid ocean currents or seasonal typhoons [51].

Findings from this study have three important ecological implications. First, northward dispersal of *L*. *semifasciata* is a real phenomenon, and has the potential to establish a new population beyond the typical distribution range. Second, relatively long-distance dispersal of *L*. *semifasciata* confirms the importance of ocean currents in the dispersal of marine animals, as recently shown in *H*. *platurus* [32]. Third, because *L*. *semifasciata* are venomous sea kraits (although they are less likely to bite) [10], appropriate education on sea snake bites is immediately necessary at the coastal towns in South Korea. Questions still remain on whether *L*. *semifasciata* in Korea originate from specific population or various populations, how long *L*. *semifasciata* survive at the new northern sites, whether new breeding populations are being established, and how this species as a new predator impact local marine ecosystems [52]. Detailed studies on *L*. *semifasciata* in Korea and other worldwide sea snake populations beyond typical the distribution range will help clarify these issues.

## Supporting information

S1 Fig Maximum likelihood (ML) tree inferred from a dataset of partial sequences of mitochondrial cytochrome b (*Cytb*) gene. The new specimens collected in our study (12 from Korea, 4 from Taiwan) are in larger, bold font. All were identified as *Laticuada semifasciata*. Numbers at the end of the taxon name refer to GenBank accession numbers. Numbers on the branches represent support values for the major groups—ML bootstrap support, followed by Bayesian posterior probabilities.
(TIFF)

S1 Table Collection information, morphological characters and mitochondrial cytochrome b (*Cytb*) haplotype of the 12 *Laticauda semifasciata* collected in Korean waters. The haplotype names of *Cytb* are based on [23].
(DOCX)
