# Supplementary figures and images for: Northward dispersal of sea kraits (Laticauda semifasciata) beyond their typical range
Source: PLoS One. 2017 Jun 23;12(6):e0179871. doi: 10.1371/journal.pone.0179871 (PMC5482473; doi:10.1371/journal.pone.0179871)

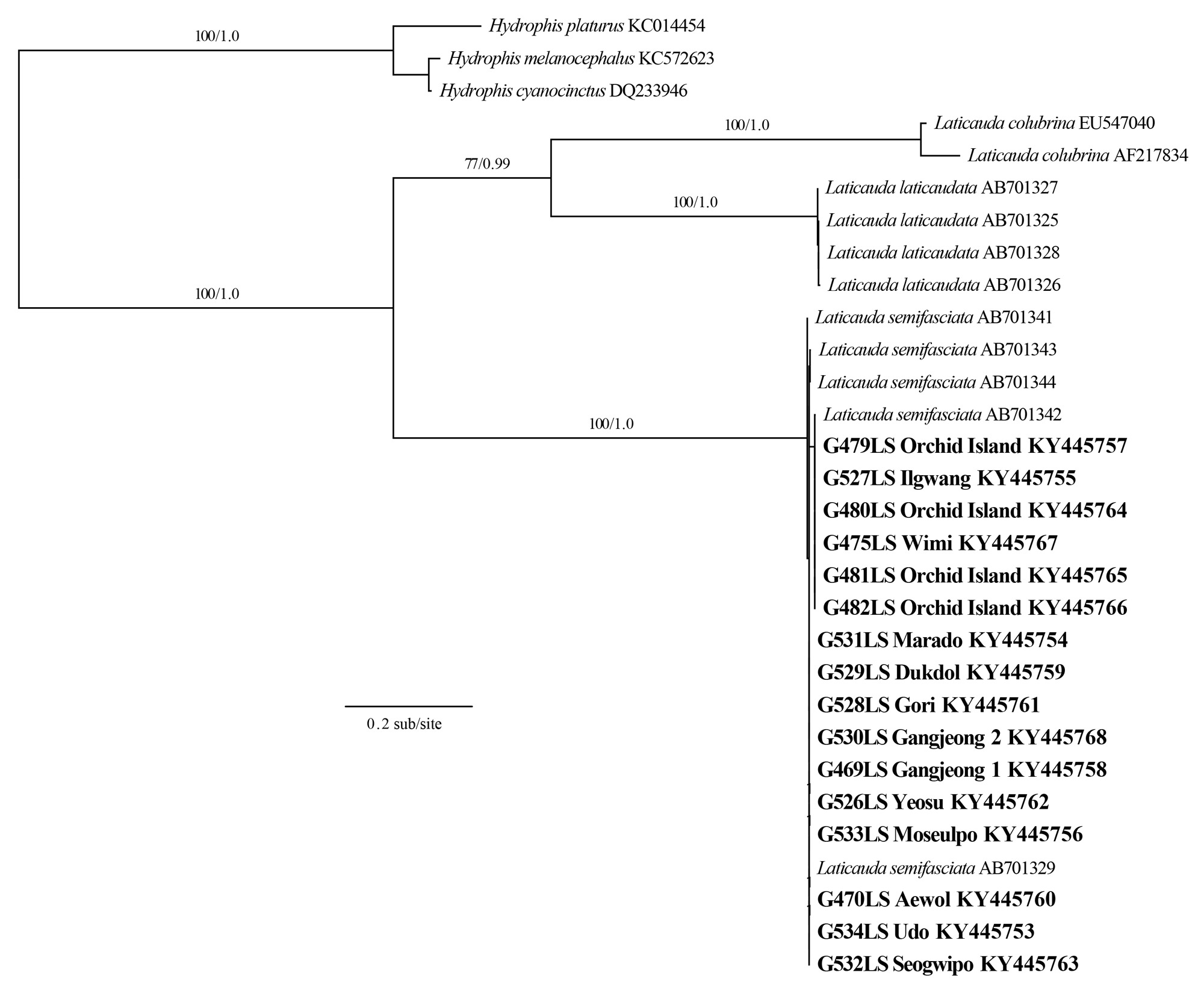

Supplement: S1 Fig — The new specimens collected in our study (12 from Korea, 4 from Taiwan) are in larger, bold font. All were identified as Laticuada semifasciata. Numbers at the end of the taxon name refer to GenBank accession numbers. Numbers on the branches represent support values for the major groups—ML bootstrap support, followed by Bayesian posterior probabilities. (TIFF) [file pone.0179871.s001.tiff]
