# Supplementary material for: Northward dispersal of sea kraits (Laticauda semifasciata) beyond their typical range
Source: PLoS One. 2017 Jun 23;12(6):e0179871. doi: 10.1371/journal.pone.0179871 (PMC5482473; doi:10.1371/journal.pone.0179871)
Supplement: S1 Table — The haplotype names of Cytb are based on [23]. (DOCX) [file pone.0179871.s002.docx]

| Voucher No. | | G469LS | G470LS | G475LS | G526LS | G527LS | G528LS | G529LS | G530LS | G531LS | G532LS | G533LS | G534LS |
| --- | --- | --- | --- | --- | --- | --- | --- | --- | --- | --- | --- | --- | --- |
| Captured date | | 2015.8.26 | 2015.9.17 | 2015.11.03 | 2016.06.23 | 2016.08.08 | 2016.08.18 | 2016.08.19 | 2016.08.26 | 2016.08.27 | 2016.08.28 | 2016.09.05 | 2016.09.07 |
| Captured location | | Gangjeong 1 | Aewol | Wimi | Yeosu | Ilgwang | Gori | Dukdol | Gangjeong 2 | Marado | Seogwipo | Moseulpo | Udo |
| GPS N | | 33°12'58.08" | 33°27'54.24" | 33°15'10" | 34°41'31.79" | 35°15'36.56" | 35°19'8.90" | 33°17'22.15" | 33°13'11.84" | 33°6'21.59" | 33°13'34.87" | 33°13'31.99" | 33°33'56.00" |
| GPS E | | 126°29'19.23" | 126°16'59.70" | 126°42'50" | 127°42'40.69" | 129°14'42.87" | 129°17'42.93" | 126°47'8.36" | 126°28'2.14" | 126°16'24.57" | 126°33'35.84" | 126°13'53.05" | 127°01'25.00" |
| Physical  Characteristics  & Sex | 1. SVL | 787 | 1030 | 965 | 1005 | 975 | 1100 | 965 | 1018 | 999 | 900 | 978 | 871 |
|  | 2. Tal | 129 | 138 | 128 | 125 | 135 | 153 | 123 | 132 | 141 | 114 | 130 | 110 |
|  | 3. TL | 916 | 1168 | 1093 | 1130 | 1110 | 1253 | 1088 | 1150 | 1140 | 1014 | 1108 | 981 |
|  | 4. BW | 841.2 | 750.9 | 736.1 | 927.1 | 712.8 | 1033 | 530 | 672 | 664.9 | 461.9 | 713 | 513.9 |
|  | 5. Sex | F | F | F | F | F | F | F | F | M | F | F | F |
| Head scale | 1. Frontal | 1 | 1 | 1 | 1 | 1 | 1 | 1 | 1 | 1 | 1 | 1 | 1 |
|  | 2. Internasal L | 1 | 1 | 1 | 1 | 1 | 1 | 1 | 1 | 1 | 1 | 1 | 1 |
|  | 3. Internasal R | 1 | 1 | 1 | 1 | 1 | 1 | 1 | 1 | 1 | 1 | 1 | 1 |
|  | 4. Nasal L | 1 | 1 | 1 | 1 | 1 | 1 | 1 | 1 | 1 | 1 | 1 | 1 |
|  | 5. Nasal R | 1 | 1 | 1 | 1 | 1 | 1 | 1 | 1 | 1 | 1 | 1 | 1 |
|  | 6. Postocular L | 2 | 2 | 2 | 2 | 2 | 2 | 2 | 2 | 2 | 2 | 2 | 2 |
|  | 7. Postocular R | 2 | 2 | 2 | 2 | 2 | 2 | 2 | 2 | 2 | 2 | 2 | 2 |
|  | 8. Parietal | 2 | 2 | 2 | 2 | 2 | 2 | 2 | 2 | 2 | 2 | 2 | 2 |
|  | 9. Prefrontal | 3 | 3 | 4 | 3 | 3 | 3 | 3 | 3 | 3 | 3 | 3 | 3 |
|  | 10. Preocular L | 1 | 1 | 1 | 1 | 1 | 1 | 1 | 1 | 1 | 1 | 1 | 1 |
|  | 11. Preocular R | 1 | 1 | 1 | 1 | 1 | 1 | 1 | 1 | 1 | 1 | 1 | 1 |
|  | 12. Rostral | 2 | 2 | 2 | 2 | 2 | 2 | 2 | 2 | 2 | 2 | 2 | 2 |
|  | 13. Supraocular L | 1 | 1 | 1 | 1 | 1 | 1 | 1 | 1 | 1 | 1 | 1 | 1 |
|  | 14. Supraocular R | 1 | 1 | 1 | 1 | 1 | 1 | 1 | 1 | 1 | 1 | 1 | 1 |
|  | 15. Supralabial L | 7 | 7 | 7 | 7 | 7 | 7 | 7 | 7 | 7 | 7 | 7 | 7 |
|  | 16. Supralabial R | 7 | 7 | 7 | 7 | 7 | 7 | 7 | 7 | 7 | 7 | 7 | 7 |
|  | 17. Infralabial L | 6 | 7 | 7 | 7 | 7 | 7 | 7 | 7 | 7 | 7 | 7 | 7 |
|  | 18. Infralabial R | 7 | 7 | 7 | 7 | 7 | 7 | 7 | 7 | 7 | 7 | 7 | 7 |
|  | 19. Temporal L | 2+3 | 2+3 | 2+3 | 2+3 | 2+3 | 2+3 | 2+3 | 2+3 | 2+3 | 2+3 | 2+3 | 2+3 |
|  | 20. Temporal R | 2+3 | 2+3 | 2+3 | 2+3 | 2+3 | 2+3 | 2+3 | 2+3 | 2+3 | 2+3 | 2+3 | 2+3 |
| Body scale | 1. Ventrals | 199 | 202 | 199 | 201 | 199 | 198 | 200 | 202 | 206 | 195 | 202 | 202 |
|  | 2. Subcaudals | 35 | 36 | 36 | 36 | 35 | 35 | 34 | 34 | 39 | 36 | 38 | 37 |
|  | 3. On the neck | 21 | 23 | 23 | 23 | 25 | 23 | 23 | 23 | 22 | 23 | 23 | 23 |
|  | 4. On the mid- body | 21 | 23 | 23 | 23 | 25 | 23 | 23 | 23 | 23 | 23 | 23 | 23 |
|  | 5. On near anus | 21 | 21 | 21 | 19 | 22 | 21 | 21 | 21 | 17 | 22 | 21 | 21 |
| Band | 1. No. on the trunk | 36 | 34 | 37 | 37 | 41 | 32 | 30 | 31 | 36 | 35 | 33 | 33 |
|  | 2. No. on the Tail | 7 | 6 | 6 | 7 | 7 | 5 | 6 | 6 | 5 | 6 | 6 | 6 |
| *Cytb* haplotype | | Semi-5 | Semi-1 | Semi-3 | Semi-5 | Semi-3 | Semi-1 | Semi-5 | Semi-1 | Semi-5 | Semi-5 | Semi-1 | Semi-5 |
